# Toward an Ecologically Optimized N:P Recovery from Wastewater by Microalgae

**DOI:** 10.3389/fmicb.2017.01742

**Published:** 2017-09-11

**Authors:** Tânia V. Fernandes, María Suárez-Muñoz, Lukas M. Trebuch, Paul J. Verbraak, Dedmer B. Van de Waal

**Affiliations:** Department of Aquatic Ecology, Netherlands Institute of Ecology (NIOO-KNAW) Wageningen, Netherlands

**Keywords:** decentralized black water treatment, algal photobioreactor, nutrient removal, *Chlorella*, nitrogen, phosphorus

## Abstract

Global stores of important resources such as phosphorus (P) are being rapidly depleted, while the excessive use of nutrients has led to the enrichment of surface waters worldwide. Ideally, nutrients would be recovered from wastewater, which will not only prevent eutrophication but also provide access to alternative nutrient stores. Current state-of-the-art wastewater treatment technologies are effective in removing these nutrients from wastewater, yet they can only recover P and often in an insufficient way. Microalgae, however, can effectively assimilate P and nitrogen (N), as well as other macro- and micronutrients, allowing these nutrients to be recovered into valuable products that can be used to close nutrient cycles (e.g., fertilizer, bioplastics, color dyes, and bulk chemicals). Here, we show that the green alga *Chlorella sorokiniana* is able to remove all inorganic N and P present in concentrated toilet wastewater (i.e., black water) with N:P ratios ranging between 15 and 26. However, the N and P uptake by the algae is imbalanced relative to the wastewater N:P stoichiometry, resulting in a rapid removal of P but relatively slower removal of N. Here, we discuss how ecological principles such as ecological stoichiometry and resource-ratio theory may help optimize N:P removal and allow for more effective recovery of N and P from black water.

## Introduction

Welcome to the world of tomorrow, where waste no longer exists. In this world waste is converted into resources that can be reused. Today, essential resources are being depleted, literally flushed down our toilets. Among the main examples is phosphorus (P), which is a major element in life for it is involved in energy transfer (ATP), cellular structures (phospholipids), and storage and transfer of genetic information (DNA/RNA) (Sterner and Elser, 2002). Human waste contributes to 68% of the total P present in domestic wastewater (Kujawa-Roeleveld and Zeeman, 2006). If we were able to recover that P, human excreta could supply 22% of the global P demand (Mihelcic et al., 2011). This is particularly important because global reserves of P are becoming increasingly scarce, expensive, and unevenly distributed, which will have important societal consequences (Elser and Bennett, 2011; Cordell and White, 2014). Human waste contains between 1.8 and 10 g P kg^-1^ (Roy, 2017), but also many valuable macro- and micronutrients. Recovery of these elements alongside P may improve fertilizer quality (de Graaff et al., 2011).

Current wastewater treatment plants can effectively remove nitrogen (N) and P from wastewater, therefore preventing the enrichment of surface waters with nutrients. Despite this removal, actual recovery of increasingly important resources, such as P, is practically non-existent. In the few cases that P is recovered, as for example by struvite precipitation, this is often inefficient (Hao et al., 2013; Egle et al., 2016). Microalgae can effectively assimilate P, but also N, and other macro-/micronutrients that are present in the wastewater, allowing these nutrients to be utilized for the production of valuable products such as biofuels, bioplastics, dyes, and bulk chemicals (Wijffels and Barbosa, 2010; Wijffels et al., 2010; Zeller et al., 2013; Suganya et al., 2016). Microalgae can also be used as an enriched fertilizer, as they include a wide range of micronutrients (Mg, Fe, Co, etc.). These micronutrients are often missing in commonly used artificial fertilizers, leading to nutrient depletion of agricultural soils (Udo de Haes et al., 2012). Moreover, microalgae fertilizer can improve soil structure and water retention capacity (Metting, 1990; Maurya et al., 2015).

Wastewater N:P ratios depend on the origin of the wastewater. Municipal wastewater, which includes domestic wastewater with minor contributions of industrial wastewater (common in industrialized countries), have N:P ratios of approximately 10 (Henze and Comeau, 2008). Wastewater with animal manure and human excreta can reach N:P ratios up to 40 (Kumar et al., 2010; Tuantet et al., 2013). In concentrated toilet wastewater (i.e., black water), the N:P ratio usually varies from 20 to 30 (Vasconcelos Fernandes et al., 2015). Earlier work has demonstrated how the microalga *Chlorella sorokiniana* can fully remove N and P from black water with 76 mmol NH_4_^+^ L^-1^ and 3.4 mmol 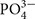, yielding an N:P ratio of 22. All P was removed from the black water in 4 days, but another 8 days were needed for all N to be removed (Vasconcelos Fernandes et al., 2015). This may be due to the high N:P ratio of the black water, as well as due to a low N relative to P removal and recovery by the microalgae. Here, we first tested how black water N:P stoichiometry can affect N:P removal and recovery ratios by the green alga *C. sorokiniana*. Additionally, we highlight how microalgae may optimize nutrient removal from wastewater by applying the ecological principles of ecological stoichiometry (Sterner and Elser, 2002) and resource-ratio theory (Tilman, 1982).

## Materials and Methods

*Chlorella sorokiniana* was cultivated in anaerobically treated black water (AnBW) with different initial N:P ratios (15, 17, 20, 23, and 26). The lower N:P ratios were achieved by addition of PO_4_^3-^. AnBW was collected from an upflow anaerobic sludge blanket (UASB) reactor fed with vacuum collected black water and operated at 35°C and a hydraulic retention time of 8 days. AnBW was thermally pre-treated at 55°C for 4 days to eliminate the interference of bacteria on C, N, and P uptake. The inoculum density of the experimental cultures was 0.5 g dry weight L^-1^. The experiment was performed in 400 mL flat panel photobioreactors (PBRs) with a light path of 14 mm. The PBRs were illuminated with a maximum incident light intensity of 1,500 μmol photons m^-2^ s^-1^, an average *in situ* light intensity of 945 μmol photons m^-2^ s^-1^, and a light:dark cycle of 16 h:8 h. Temperature was set at 37°C and controlled by a water jacket placed between the light source and bioreactor. The pH was kept at 6.8 ± 0.1 by automated acid/base additions to prevent the conversion of ammonium ions (NH_4_^+^) to gaseous ammonia (NH_3_), which is harmful to green microalgae (Abeliovich, 2005). The culture was mixed by aeration with compressed air (enriched with 10% CO_2_) at a flow of 400 mL min^-1^. Daily samples were analyzed for biomass and dissolved inorganic nutrients. Particulate C, N, and P were assessed at day 4 and at the end of each treatment (between days 9 and 18). Biomass was determined by dry weight. NH_4_^+^ and PO_4_^3-^ were analyzed by a Seal QuAAtro Auto Analyzer (Seal Analytical Inc., Netherlands). Particulate N and C were measured on a FLASH 2000 NC Elemental Analyzer (Brechbuhler Incorporated, Interscience B.V., Netherlands). Particulate P was first combusted at 550°C for 30 min, then digested with persulfate (2.5%) at 121°C for 30 min, and subsequently analyzed on the Seal QuAAtro Auto Analyzer. The ratio at which N and P were removed from the black water was calculated from the linear relation between dissolved inorganic N and P concentrations over the first 4 days of the experiment.

## Results and Discussion

**FIGURE 1:**
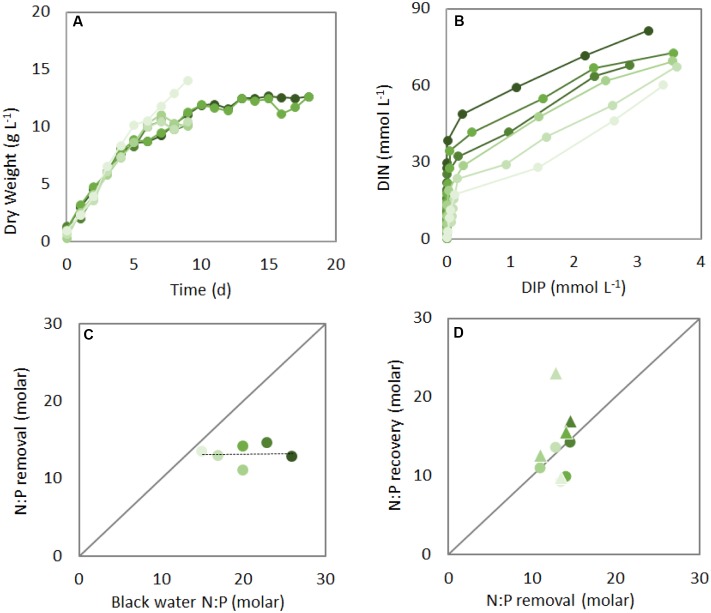
Dynamics of biomass **(A)**, dissolved inorganic nitrogen (DIN) and phosphorus (DIP) **(B)**, N:P removal **(C)**, and N:P recovery **(D)** by *Chlorella sorokiniana* at different initial black water N:P ratios (green shades, darker colors represent higher N:P). Symbols in **(A)** and **(B)** indicate daily measurements. In **(B)** the highest N and P concentrations of the beginning of the experiment are at the top right corner. Symbols in **(C)** indicate the integrated N:P removal rates during the first 4 days of each experiment, and circles and triangles in **(D)** show the cellular N:P after the first 4 days and the end of the experiment, respectively.

Algal biomass increased similarly in the different black water N:P ratio treatments, reaching stationary phase in about 10 days (**Figure 1A**). Almost all dissolved inorganic N and P were removed via algal assimilation, showing similar dynamics irrespective of initial wastewater N:P ratios, with full removal of P within 4 days and subsequent removal of remaining N in another 5–10 days (**Figure 1B** and Supplementary Figure 1). The N:P removal ratio, i.e., the ratio at which N and P were removed from the black water, was about 13 during the first 4 days of each treatment (i.e., the P replete phase), and lower than the initial black water N:P (**Figure 1C**). The N:P recovery, i.e., the cellular N:P of *Chlorella*, largely followed the N:P removal during the initial 4 days (see circles in **Figure 1D**). During the subsequent P deplete phase, algae continued to grow for another 3–6 days, which lead to increased N:P ratios at the end of each treatment compared to day 4 (compare triangles to circles in **Figure 1D**). An increase in cellular N:P as well as C:P ratios upon P limitation is commonly observed in phytoplankton (Klausmeier et al., 2004a; Hillebrand et al., 2013), and is caused by a continued uptake and assimilation of N and C after P is limited (Sterner and Elser, 2002; Supplementary Figure 2).

Our results show that both the initial N:P removal and N:P recovery ratio are independent of the initial black water N:P ratios, and demonstrate further cellular uptake and removal of N after P has been depleted from the medium. Overall, the observed biomass increase and nutrient removal from black water were very effective, with a final mean biomass yield of 12 g dry weight L^-1^ (**Figure 1A**), final mean biomass C:N:P ratio of 125:14:1, and mean biomass yield per light photon of 0.20 g dry weight (mol photon)^-1^. The recovery efficiencies of N and P from the black water by algal assimilation were 75 and 100%, respectively. The lower recovery of N is presumably due to volatilization of N as NH_3_ and N_2_O (Fagerstone et al., 2011; Mezzari et al., 2013) and release of dissolved organic nitrogen compounds by the microalgae (Sipler and Bronk, 2015). Thus, all P and most of the N from the wastewater were converted into algal biomass.

The average nutrient removal rates over the period of P-sufficiency to P-depletion (i.e., day 0 to day 4) were 7.6 mmol N L^-1^ d^-1^ and 0.9 mmol P L^-1^ d^-1^, respectively. Compared to an earlier study with continuous light, the observed yield of biomass on light, N removal rates, and P removal rates increased by about 25, 34, and 81%, respectively. This suggests that despite the high microalgal biomass and associated self-shading, the availability of light was not limiting and a shorter light:dark cycle even promoted a higher biomass yield on light. These findings support the application of microalgae for wastewater treatment using natural light with day:night cycling. The delayed removal of N relative to P, however, imposes a great challenge to the application of microalgae for treatment of wastewater with high N:P ratios. For instance, on a small-scale decentralized black water treatment system for ∼250 people where only 1 L of flush water is used, a 5-day delayed N removal is associated with a black water accumulation of 2–10 m^3^ for each treatment cycle (depending on toilet usage – office or household). In practical terms, such a delay would lead to the need of a large AnBW storage capacity, which adds complexity to the treatment system. Thus, higher N:P removal and consequently N:P recovery ratios are essential for a more effective use of microalgae in wastewater treatment.

**FIGURE 2:**
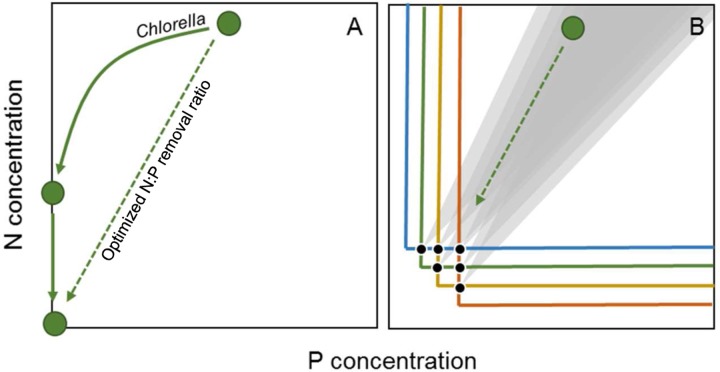
Schematic representation of current N and P removal with *Chlorella* **(A)** and expected removal with multiple species in a continuous culture system **(B)**, where horizontal and vertical lines indicate zero net growth isoclines or *R*^∗^ values, with colors denoting different species and the black circles indicating equilibrium points where two species can coexist. The gray areas in **(B)** indicate hypothesized ranges in consumption vectors, or N:P uptake ratios, of species coexisting at the associated equilibrium point. The diagonal dashed arrows indicate an optimized higher N:P removal ratio.

Previous studies have shown how ecological approaches (e.g., complementarity in resource use) may support industrial scale biomass, crop, and diesel production (Smith et al., 2010; Stockenreiter et al., 2012; Shurin et al., 2013). Comparable principles will apply to the removal and recovery of nutrients from wastewater. The rate at which nutrients can be removed and recovered from the wastewater is directly associated to key physiological traits of alga species, such as growth rate and nutrient demands. Higher growth rates are generally associated with higher nutrient uptake rates, and thereby result in faster removal of nutrients from wastewater. Yet, algal species with high growth rates may also have higher P demands, and thereby lower N:P ratios (Geider and LaRoche, 2002; but see Flynn et al., 2010). Thus, although selection of single species with higher growth rates may enhance the overall removal rate of nutrients, it may not enhance the N:P removal ratio. On the other hand, selection of a single species with N:P uptake ratios resembling the N:P of the wastewater may accelerate the combined removal of N and P (**Figure 2A**). In our study, the cellular microalgal N:P ratio was 13 during the P replete phase of the experiment, which was lower compared to the black water. Phytoplankton species with higher optimal N:P ratios [i.e., N:P ratios under nutrient sufficient maximum growth rates (Klausmeier et al., 2004b)] should lead to higher N:P removal ratios and overall N removal rates. The black water N:P ratios are at the higher end of reported optimal phytoplankton N:P ratios (Geider and LaRoche, 2002), and are found in some species of cyanobacteria and green algae (Hillebrand et al., 2013). Thus, further screening of particularly these groups may provide the best chances at finding species with N:P ratios resembling that of black water and thus optimizing N:P removal and recovery from wastewater (**Figure 2A**).

Besides optimized N:P removal by selection of a single species, enhanced N:P removal and N:P recovery ratios could also be achieved using phytoplankton communities, where each species has a complementary N:P uptake ratio. In competition for two nutrients at steady state in a continuous culture system (e.g., a chemostat), trade-offs may lead to stable coexistence of two species, where both resources are depleted to the minimum requirements of either species (Tilman, 1982). This minimum required is indicated by *R*^∗^, and gives the amount of a resource where growth and losses are balanced (i.e., zero-net-growth-isoclines, **Figure 2B**). Thus, growth will be positive (biomass will increase) at concentrations above the *R*^∗^ value, while growth will be negative (biomass will decrease) at concentrations below *R*^∗^. The *R*^∗^ for a nutrient will generally be lower for species with a high affinity (i.e., a low half-saturation constant K_1/2_) and high uptake rate for a particular nutrient (Litchman et al., 2007). For instance, if one species has a high affinity and uptake rate for N, it will likely have a low *R*^∗^ for N as well, and will be a better competitor for N. A trade-off between competitive abilities for N and P between different species may facilitate coexistence at intermediate nutrient supply ratios, where neither species can competitively exclude the other (**Figure 2B**).

The chance for coexistence, and thereby optimized removal of both N and P, will increase with the number of species exhibiting trade-offs between the competitive abilities for N and P. Indeed, although mixtures of multiple algal species showed differential effects on algal production (Schmidtke et al., 2010; Shurin et al., 2013, 2014), enhanced diversity did show reduced residual concentrations of nutrients in various systems (Cardinale et al., 2006; Shurin et al., 2013) and may support higher nutrient use efficiencies (Ptacnik et al., 2008), and overall nutrient uptake rates (Cardinale, 2011). Thus, an enhanced functional trait diversity in algal mixtures, and thereby a higher complementarity in nutrient use, may favor the increased removal of nutrients compared to single algal cultures.

Mixtures of species with complementary light harvesting strategies may allow a more effective use of the light spectrum. For instance, green and red cyanobacterial species were shown to coexist in white-light (Stomp et al., 2004). Thus, co-culturing of distinct green algae and cyanobacteria together with red algal species (e.g., *Haematococcus*) would likely enhance the N:P removal and N:P recovery ratio, and further increase the biomass yield from wastewater. Moreover, a higher algal diversity may enhance the resilience of a system against variations in growth conditions, top-down control, and pathogen infections (Shurin et al., 2013).

Application of ecological principles for technological microalgal application is still in its infancy, particularly for wastewater treatment. Here, we highlighted how our understanding of trade-offs and complementarity in resource acquisition and demands may support optimized N:P removal ratios and ultimately greater recovery of nutrients from wastewater. Connecting wastewater nutrient reuse to product cycles will support a more sustainable future, where a full understanding of phytoplankton eco-physiology provides an overarching guide to effective nutrient removal, recovery, and the production of valuable algal biomass-based products from wastewater.

## Author Contributions

TF, MS-M, and DW did the conception of the work; LT and PV performed the experimental work; TF supervised the experimental work; TF and DW wrote a first draft of the manuscript, which was revised by all co-authors.

## Conflict of Interest Statement

The authors declare that the research was conducted in the absence of any commercial or financial relationships that could be construed as a potential conflict of interest.
