# Supplementary material for: Toward an Ecologically Optimized N:P Recovery from Wastewater by Microalgae
Source: Front Microbiol. 2017 Sep 11;8:1742. doi: 10.3389/fmicb.2017.01742 (PMC5601980; doi:10.3389/fmicb.2017.01742)
Supplement: Supplementary file 1 [file Image_1.PDF]

Supplementary Material

Towards an ecologically optimized N:P recovery from wastewater by microalgae

Tânia V. Fernandes[1*], María S. Muñoz[1], Lukas M. Trebuch[1], Paul J. Verbraak[1], Dedmer B. Van de Waal[1]

[1] Department of Aquatic Ecology, Netherlands Institute of Ecology (NIOO-KNAW), Droevendaalsesteeg 10, 6708 PB Wageningen, The Netherlands. (E-mail: *T.Fernandes@nioo.knaw.nl*)

*** Correspondence:**

Tânia V. Fernandes

T.Fernandes@nioo.knaw.nl

1.1 Supplementary Figures

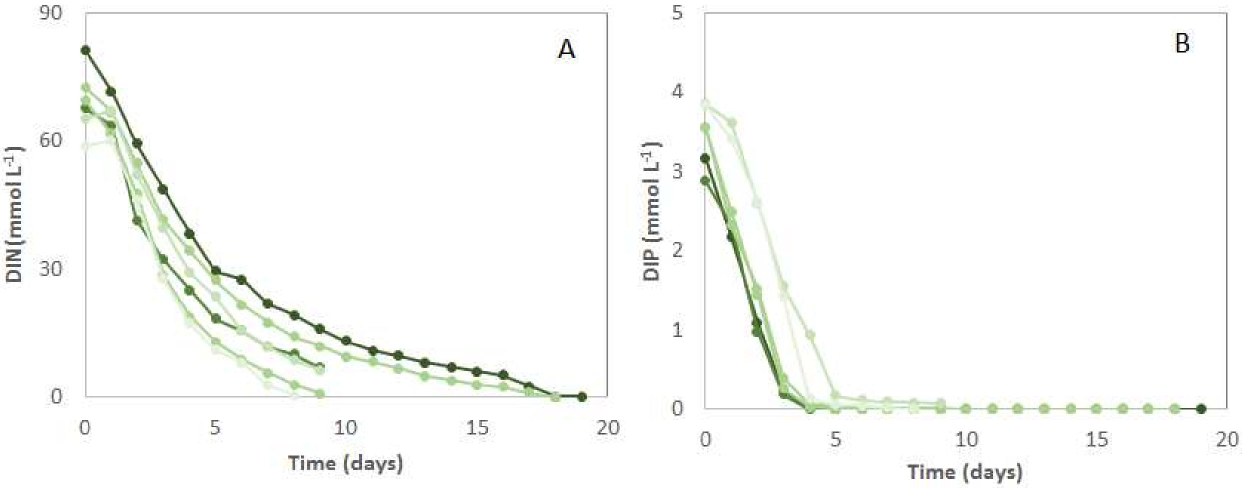

Supplementary Figure 1. Dynamics of DIN (A) and DIP (B) at different initial black water N:P ratios (green shades, darker colors represent higher N:P).

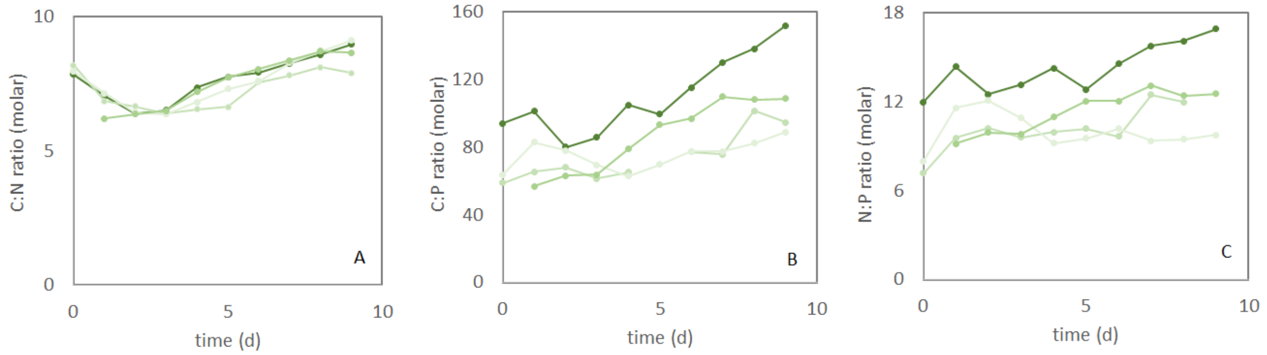

Supplementary Figure 2. Dynamics of C:N (A), C:P (B) and N:P ratios (C) of *Chlorella sorokiniana* at different initial black water N:P ratios (green shades, darker colors represent higher N:P).
